# Thymoquinone attenuates tumor growth in Apc^Min^ mice by interference with Wnt-signaling

**DOI:** 10.1186/1476-4598-12-41

**Published:** 2013-05-13

**Authors:** Michaela Lang, Melanie Borgmann, Georg Oberhuber, Rayko Evstatiev, Kristine Jimenez, Kyle W Dammann, Manuela Jambrich, Vineeta Khare, Christoph Campregher, Robin Ristl, Christoph Gasche

**Affiliations:** 1 Department of Medicine III, Division of Gastroenterology and Hepatology, Medical University of Vienna, Vienna, Austria; 2 Christian Doppler Laboratory for Molecular Cancer Chemoprevention, Medical University of Vienna, Vienna, Austria; 3 Pathology Ueberlingen, Ueberlingen, Germany; 4 Center for Medical Statistics, Informatics, and Intelligent Systems, Section for Medical Statistics, Medical University of Vienna, Vienna, Austria

**Keywords:** *Nigella sativa*, Thymoquinone, Chemoprevention, Colon cancer, Familial adenomatous polyposis

## Abstract

**Background:**

Patients with familial adenomatous polyposis (FAP) are at increased risk for the development of colorectal cancer. Surgery and chemoprevention are the most effective means to prevent cancer development. Thymoquinone (TQ) is considered the main compound of the volatile *Nigella sativa* seed oil and has been reported to possess anticarcinogenic properties. In this study we evaluated the chemopreventive properties of TQ in a mouse model of FAP.

**Methods:**

APC^Min^ mice were fed with chow containing 37.5 mg/kg or 375 mg/kg TQ for 12 weeks. H&E stained intestine tissue sections were assessed for tumor number, localization, size, and grade. Immunohistochemistry for β-catenin, c-myc, Ki-67 and TUNEL-staining was performed to investigate TQ’s effect on major colorectal cancer pathways. TQ’s impact on GSK-3β and β-catenin were studied in RKO cells.

**Results:**

375 mg/kg but not 37.5 mg/kg TQ decreased the number of large polyps in the small intestine of APC^Min^ mice. TQ induced apoptosis in the neoplastic tissue but not in the normal mucosa. Furthermore, upon TQ treatment, β-catenin was retained at the membrane and c-myc decreased in the nucleus, which was associated with a reduced cell proliferation in the villi. *In vitro*, TQ activated GSK-3β, which induced membranous localization of β-catenin and reduced nuclear c-myc expression.

**Conclusions:**

In summary, TQ interferes with polyp progression in Apc^Min^ mice through induction of tumor-cell specific apoptosis and by modulating Wnt signaling through activation of GSK-3β. *Nigella sativa* oil (or TQ) might be useful as nutritional supplement to complement surgery and chemoprevention in FAP.

## Background

In developed countries, colorectal cancer is one of the most common types of cancer, ranking 2^nd^ and 3^rd^ in women and men respectively. The lifetime risk for colorectal cancer development in the general population is approximately 6% and is responsible for about 8% of all cancer deaths worldwide [1]. Certain familial syndromes are at increased risk for the development of colorectal cancer. This is about 80% in hereditary nonpolyposis colorectal cancer and almost 100% in familial adenomatous polyposis (FAP) [2].

FAP is an autosomal dominantly inherited disease caused by a germline mutation of the adenomatous polyposis coli gene (APC) located on chromosome 5q21 [3,4]. It is characterized by the development of numerous, predominantly large intestinal adenomatous polyps, ultimately leading to colorectal cancer [2]. Besides endoscopic surveillance and prophylactic surgery, chemoprevention is the most effective means to repress cancer development in FAP. Inhibition of polyp progression improves tumor outcome, thereby prolonging survival in FAP patients. The protective effect of non-steroidal anti-inflammatory drugs (NSAIDs) such as aspirin [5], sulindac [6,7] and the selective cyclooxygenase-2 inhibitor celecoxib [8] has been extensively studied in FAP patients. Some also reduce rectal polyp burden after colectomy with ileorectal anastomosis [6]. Celecoxib was shown to be beneficial against the duodenal tumor burden [9]. However, a chemoprevention study dealing with genotypically but not phenotypically affected patients treated with sulindac failed to show prevention of primary adenoma development [7]. Unfortunately, these drugs are associated with gastrointestinal (bleeding, ulceration) and cardiovascular side effects (thromboembolic events and heart failure) in the long-term [10-12].

In consideration of such side effects, the use of natural substances such as curcumin, eicosapentaenoic acid, apple polyphenols, capsaicin, or thymoquinone (TQ) for chemoprevention is emerging [13-15]. TQ is the main active component of the volatile *Nigella sativa* (black cumin) seed oil, which is used as a spice in countries with low incidence of colorectal cancer such as Egypt, Pakistan, or India. Traditional medicine has utilized its anti-inflammatory, antioxidant, and anti-carcinogenic properties, supporting TQ as a promising dietary chemopreventive agent [16]. *In vitro* studies indicate that TQ inhibits tumor cell proliferation in various cancers [17-19], including colorectal cancer [20,21]. TQ induces a G1 cell cycle arrest, increases p53 and p21WAF1 protein levels, induces apoptosis in a dose- and time-dependent manner, and reduces Bcl-2 protein in HCT116. Further actions of TQ include inhibition of angiogenesis, endothelial cell migration, invasion, and tube formation as demonstrated in HUVECs [18]. *In vivo* weekly i.p. injections of 5mg/kgbw TQ reduced the number and size of aberrant crypt foci and tumor multiplicity in a chemically-induced colorectal cancer mouse model. The suppression of tumor development was sustainable, as treatment with TQ resulted in a reduction of tumor number even after a 10-week discontinuation. Furthermore, in a HCT116 cell xenograft model, a 3-times weekly i.p. injection of 20 mg/kg TQ reduced the relative tumor size by 29% from 2.8 to 2.0 mm^2^[20]. This study was designed to test the chemopreventive effect of TQ in Apc^Min^ (APC, adenomatous polyposis coli; Min, multiple intestinal neoplasia) mice, which best resemble the FAP phenotype.

## Results

### TQ attenuates tumor growth in Apc^Min^ mice

To evaluate the effect of TQ on polyp formation in the APC^Min^ mouse, 4–6 week old female and male animals were randomly divided into 4 groups and treated over a period of 12 weeks. Neither TQ nor piroxicam influenced weight gain and food uptake (Additional file 1: Figure S1). Mouse colonoscopy at week 9 demonstrated a significant reduction of distal large intestinal polyps in the TQ-low and the piroxicam group (p<0.05), with a trend also for TQ-high (p=0.124; Additional file 2: Figure S2).

**Figure 1:**
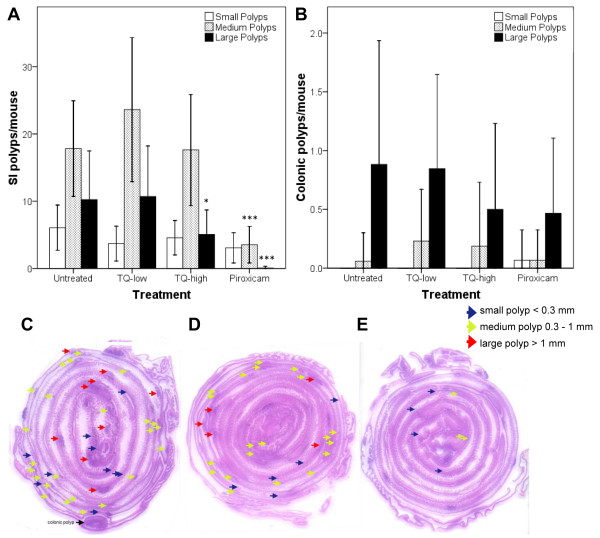
**TQ-high reduces large tumors in the small intestine (SI).** Size distribution of polyps in the SI (**A**) and colon (**B**) of APC^Min^ mice (small <0.3 mm; medium: 0.3–1 mm; large: >1 mm). Bars show mean number (± SD) of SI or colonic polyps/mouse. TQ-high (n=16) but not TQ-low (n=13) decreased the number of large polyps in the SI. Piroxicam (n=15) decreased the number of medium and large SI polyps. TQ-high and piroxicam show a trend for reduction of colonic polyps (**B**) *p<0.05, ***p<0.001; ANOVA, Dunnett 2-sided. Representative H&E-stained Swiss rolls from different treatment groups: untreated (**C**, n=17), TQ-high (**D**) and piroxicam (**E**). Arrows indicate SI polyps of different size.

At 12 weeks mice were euthanized and intestinal Swiss rolls were analyzed for tumor number, size (Additional file 1: Figure S1C), and localization (colonic or small intestine). TQ-high decreased the number of large polyps (>1mm) in the small intestine from 10 (95% CI 8–13) to 5 (2–8; p<0.05), while small and medium-sized polyps were unchanged (Figure 1). Tumor multiplicity changed minimally, from 34 (29–40) in untreated APC^Min^ mice and 38 (32–44) in TQ-low to 27 (21–33) in TQ-high mice (p= 0.22; Additional file 2: Figure S2C). Piroxicam decreased medium-sized polyps from 18 (14–22) to 4 (0–7), large polyps from 10 (8–13) to 0 (-3-3) and tumor multiplicity from 34 (29–40) to 7 (1–13) as expected (Figure 1). Colonic polyp numbers revealed no significant differences between the treatment groups (Figure 1B). A trend was observed for the reduction of colonic polyps within the piroxicam and TQ-high treated groups. Adenocarcinoma formation in the small intestine, defined as penetration of the muscularis mucosae, was found in 1 out of 13 mice in the TQ-low group and in 1 out of 16 mice in the TQ-high group (Additional file 1: Figure S1E).

### TQ induces apoptosis in polyps of Apc^Min^ mice

**Figure 2:**
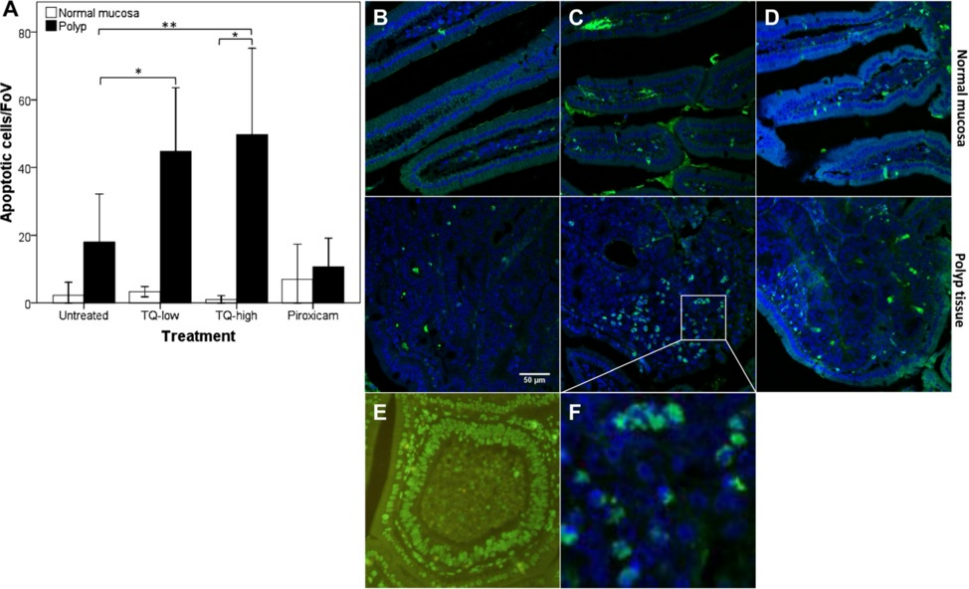
**TQ induces tumor-specific apoptosis in APC**^**Min **^**mice.** TQ-low and TQ-high significantly increased the number of apoptotic cells (by TUNEL assay) within SI polyps but not in the normal mucosa (**A**). Bar graphs display the mean number (± SD) of apoptotic cells per field of view (FoV, n=12). *p<0.05, **p<0.01; by ANOVA (2-sided Dunnett); paired T-test was used to compare the normal mucosa to neoplastic cells within each group (only significant for TQ-high). Representative images of normal mucosa (upper panel) and polyp tissue (lower panel) in untreated (**B**), TQ-high (**C, F**), and piroxicam treated APC^Min^ mice (**D**). DNase I treated positive control (**E**). DAPI (blue) and incorporated fluorescein-12-dUTP (green) signals were imaged at 400x using a confocal fluorescence microscope (LSM 5 Exciter; analysis software: LSM image examiner, Zeiss, Jena, Germany).

To ascertain the effect of TQ on apoptosis, TUNEL-staining of Swiss rolls was performed. Apoptotic cells were analyzed within polyps and normal mucosa of the small intestine. The number of apoptotic cells increased in the neoplastic but not in the normal tissue upon TQ treatment (Figure 2). The average number of apoptotic cells within polyps was 18±16 per FoV for untreated, 45±18 for TQ-low and 50±30 for TQ-high treated mice (p<0.05 and p<0.01, respectively). However, this effect was not observed in the piroxicam treated group (13±14 per FoV). These results suggest that TQ reduces polyp growth through selective induction of apoptosis.

### TQ reduces proliferation in the villi of Apc^Min^ mice

Nuclear Ki-67 staining was assessed to investigate the effect of TQ on cell proliferation. The percentage of Ki-67 positive cells was scored in polyps, normal crypts and villi of APC^Min^. Neither TQ nor piroxicam altered the number of Ki-67 cells in polyps or crypt cells (Additional file 3: Figure S3). In C57BL/6 wt mice Ki-67 positive cells are specifically restricted to the crypt cells. In APC^Min^ mice, however, we observed nuclear Ki-67 staining also in cells of the intermediate zone of the villi. A reduction of Ki-67 positive cells in TQ-high and to a lesser extent also in TQ-low treated mice was observed. The mean Ki-67 IRS in the villi was reduced from 4.8±1.6 to 4.0±1.7 in TQ-low and to 3.6±1.8 in TQ-high (p<0.05) treated cells. Again piroxicam had no effect (IRS 5.1±1.7).

### TQ reduces c-myc expression in the polyps of Apc^Min^ mice

**Figure 3:**
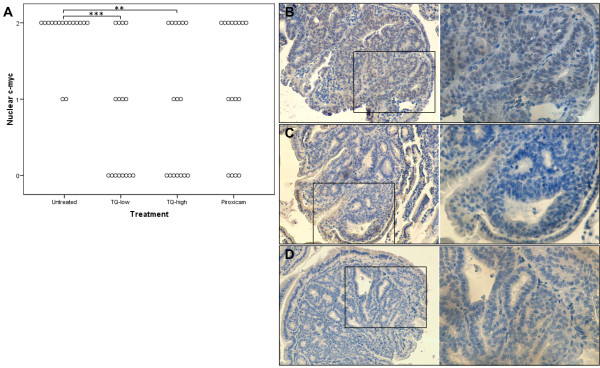
**TQ decreases nuclear c-myc protein in polyps of APC**^**Min **^**mice.** IRS of nuclear c-myc expression from polyps (n=16) of untreated, TQ-low, TQ-high, or piroxicam treated APC^Min^ mice (n=4; **A**). ANOVA, Dunnett, 2-sided was used to compare the different treatment groups to the untreated group; **p<0.01, ***p<0.001. Every dot in the graph represents a single polyp analyzed for nuclear c-myc intensity as follows: 0, negative staining; 1+, weak staining; 2+, moderate staining. Treatment with TQ-low or TQ-high significantly decreased nuclear c-myc within the polyps. Representative image for untreated (**B**) and TQ-high treated (**C**) mice. Image **D** shows the negative control sample without the primary antibody (**D**). Magnification: 100× (left panel), 200× (right panel).

We further analyzed nuclear c-myc expression, which is highly abundant in proliferative tissue and necessary for cell cycle progression [22]. TQ-low (p<0.001) and TQ-high (p<0.01) treatment reduced nuclear c-myc protein in the polyps, an effect which was not observed in piroxicam-treated mice (Figure 3).

### TQ translocates β–catenin to the membrane in APC^Min^ polyps

**Figure 4:**
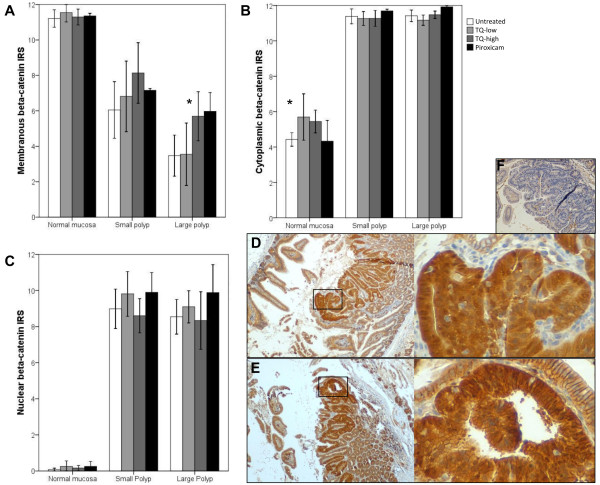
**β-catenin expression in untreated, TQ-low, TQ-high, or piroxicam treated APC**^**Min **^**mice (n=8).** Membranous (**A**), cytoplasmic (**B**), and nuclear (**C**) β-catenin IRSs were calculated for epithelial cells of the normal mucosa, small and large polyps. Bar graphs display mean β-catenin IRSs ± SD. TQ-high treated mice (**E**) showed an increase in membranous β-catenin expression in large polyps (ANOVA, Dunnett, 2-sided, *p<0.05) and a trend for an increase in small polyps (p=0.064) when compared to untreated (**D**) APC^Min^ mice. Membranous β-catenin expression in piroxicam treated mice did not reach significance (p=0.094) due to a too small number of large polyps, although a membranous relocalization of the protein was present as in the TQ-high treated mice. Cytoplasmic expression of β-catenin increased upon TQ-low and TQ-high (p=0.064) treatment in the normal mucosa. Magnification: 40x (left panel) and 400x (right panel). Panel F shows the negative control sample without the primary antibody (**F**).

**Figure 5:**
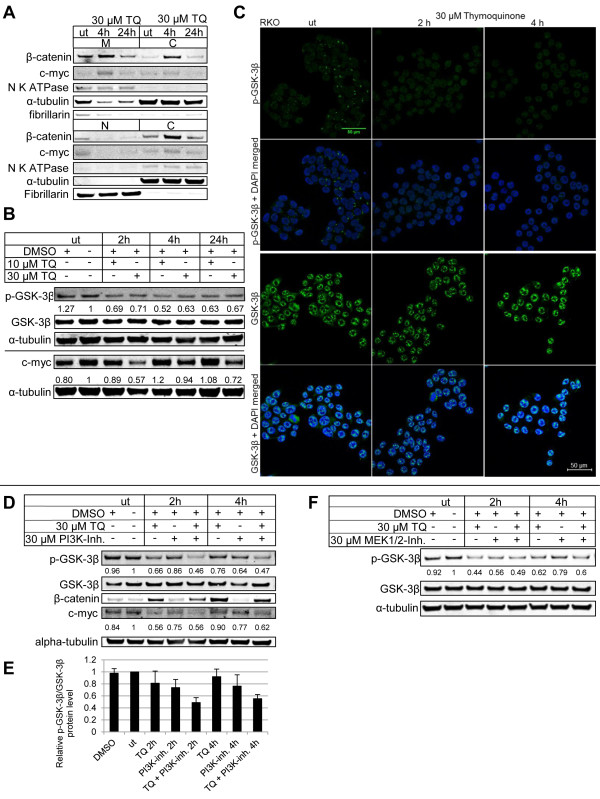
**Effect of TQ on β-catenin and the glycogen synthase kinase 3β (GSK-3β) pathway in RKO.** Separating for membranous, cytoplasmic and nuclear protein fractions showed reduction of nuclear β-catenin and c-myc upon 30 μM TQ for up to 24 h and a short term increase of membranous and cytoplasmic β-catenin. α-tubulin is used as a total protein and cytoplasmic marker, fibrillarin as nuclear marker, and Na-K-ATPase as membranous marker (**A**). TQ reduces the amount of p-GSK-3β Ser9, the inactive form of the protein, at concentrations of 10 and 30 μM, shown by Western blot (**B**) and fluorescence microscopy (**C**; top panel, green: p-GSK-3β, blue: DAPI; LSM 700). Total GSK-3β protein levels are constant over time (**B**; **C**, bottom panel). Total c-myc levels were reduced at 30 μM TQ up to 24 h, an effect that was not seen for 10 μM (**B**). 30 μM TQ and 30 μM PI3K inhibitor LY294002 additively reduced the amount of p-GSK-3β in total protein lysates. 5 independent Western blots had been carried out and the ratio of p-GSK3-3β levels and total GSK3-3β protein has been calculated with densitometry analysis (**D**, **E** and see also Table 1 for the statistical calculations). Treatment with TQ increased total β-catenin protein but reduced the amount of c-myc protein (**D**). Inhibition of MEK1/2 with 30 μM UO126 reduced p-GSK-3β, while concomitant incubation with TQ did not lead to an additive reduction of p-GSK-3β (**F**).

As TQ influenced tumor size and and c-myc expression, we considered how TQ may be implicated in the β–catenin pathway. Loss of APC protein leads to a deregulated WNT/β–catenin pathway, as APC is part of the β–catenin destruction complex. Deregulated β–catenin degradation leads to an accumulation of free β–catenin and nuclear translocation. In the nucleus, β–catenin acts as a transcription factor binding in a complex with TCF/LEF to DNA enhancer sequences leading to the upregulation of genes like the proto-oncogene c-myc [23]. A membranous, cytoplasmic, and nuclear IRS was calculated for β–catenin in the normal mucosa, small and large polyps. Remarkably, β–catenin was translocated to the membrane in large polyps of TQ-high treated APC^Min^ mice (p<0.05; Figure 4). A trend towards this effect was seen for small polyps as well (p=0.064). We found a similar trend for β–catenin shifting to the membrane in piroxicam treated mice (p=0.094). No change in β–catenin expression within the normal epithelium was identified, except for a slight increase in cytoplasmic β–catenin levels in TQ-low treated mice, an effect which was abrogated in small and large polyps. To further investigate β-catenin expression upon TQ treatment, we studied the poorly differentiated colon cancer cell line RKO, which harbors wt APC [24], wt p53 [25] and wt β-catenin alleles, the latter being expressed at low levels [24]. Treatment with TQ resulted in decreased nuclear β-catenin within 4 h and up to 24 h (as shown by Western blot). In parallel, membranous and cytoplasmic β-catenin increased at 4 h (Figure 5A). In conclusion, TQ reduces nuclear β–catenin and translocates β–catenin to the membrane.

### TQ exerts different effects on cell viability on colon cancer cells with various mutational backgrounds

To assess the effect of TQ on cell viability, MTT assays were performed in different human colon cancer cell lines (having various mutational backgrounds) and in HCEC-1CT normal diploid human colon epithelial cells. APC-truncated and p53-mutated cells (DLD1 and HT29) are the most resistant to TQ (IC_50_: 196 μM and 160 μM, respectively). LoVo, which has wt p53 and mutated APC, was the most sensitive, having an IC_50_ value of 36 μM. In comparison, cells with wt p53 and wt APC such as HCT116, RKO and HCEC-1CT were less affected by TQ, with IC_50_ values of 118, 86 and 79 μM, respectively (Additional file 4: Figure S4A). These results indicate that TQ’s effect on cell viability may be influenced by the mutational status of APC and p53. To provide further evidence that TQ induces cell apoptosis rather than cell cytostasis, RKO cells were incubated with increasing TQ concentrations for 24 h and Annexin V/PI staining was performed. Indeed, TQ treatment led to a concentration dependent increase in apoptotic and dead cells measured by flow cytometry (Additional file 5: Figure S5).

### TQ acts on the GSK-3β pathway

TQ was reported to inhibit proliferation and angiogenesis by suppressing ERK and AKT phosphorylation in HUVECs [18]. In RKO, ERK1/2 and AKT1 are highly phosphorylated. In our experiments, TQ did not have any effect on the phosphorylation status of ERK1/2 (Thr202/Tyr204) nor AKT1 (Ser473) from 30’ to 12h, despite successful inhibition of phosphorylation of ERK1/2 by the MEK1/2 inhibitor UO126 (Additional file 4: Figure S4C and D). A well-known downstream target of the PI3K/AKT pathway and, alternatively, the RAS/RAF/MEK/ERK pathway, is the glycogen synthase kinase 3β (GSK-3β). The activity of GSK-3β is inversely correlated with its phosphorylation status at Ser9. When testing for GSK-3β phosphorylation upon TQ treatment, we observed a reduction of p-GSK-3β. GSK-3β appears to be an important molecular target of TQ, which may subsequently affect the stability of c-myc (Figure 5).

### TQ downregulates c-myc protein expression

Active GSK-3β is assumed to be an important kinase phosphorylating c-myc on Thr58 for subsequent ubiquitination by the F-box protein Fbw7 [26], a component of the SCF-class ubiquitin ligase (E3) complex, and degradation. Indeed, treatment with TQ reduces c-myc expression at 2 h and 24 h (Figure 5A-C). The subcellular distribution of c-myc showed reduced expression of nuclear c-myc upon TQ treatment (Figure 5A), which is in line with the reduced nuclear c-myc expression in polyps of APC^Min^ mice (Figure 3). To further support the mediation of c-myc reduction at protein level, c-myc mRNA was quantified. In fact, the expression of c-myc mRNA was not altered upon TQ treatment (Additional file 4: Figure S4B).

### TQ reduces GSK-3β phosphorylation via inhibition of the MEK1/2 pathway rather than PI3K

As a next step we verified if the reduced GSK-3β phosphorylation upon TQ treatment was dependent on the PI3K pathway, which is upstream from GSK-3β. We used the PI3K inhibitor LY294002 together with TQ to test for synergistic or additive effects. Treatment with TQ and LY294002 for 2 h and 4 h reduced the levels of GSK-3β phosphorylation in an additive manner (see also Figure 5D, E, Table 1 and Methods section for statistical explanation) indicating that TQ’s effect on GSK-3β phosphorylation is independent of PI3K. As expected, total GSK-3β levels remained stable over time. Also, total c-myc protein was reduced more effectively at the 4 h point in time, if both substances have been added (Figure 5D).

**Table 1:** GSK-3β dephosphorylation

**Treatment**		**Experiment**					**Experiment 1-5**
		**1**	**2**	**3**	**4**	**5**	**AVG of [**[1]**] -[**[2]**] (±SD)**
DMSO		1.06	0.91	0.96			
ut		1.00	1.00	1.00	1.00	1.00	
2 h	TQ	0.90	0.87	0.66	0.47		
	PI3K-inh.	0.69	0.84	0.86	0.57		
	[1]**TQ + PI3K-inh.**	**0.61**	**0.47**	**0.46**	**0.41**		
[2]**Multiplication of TQxPI3K-Inh.**		**0.61**	**0.73**	**0.56**	**0.27**		
**Difference**[1]**-**[2]**at 2 h**		**−0.01**	**−0.26**	**−0.10**	**0.15**		−0.06 (±0.17)*
4 h	TQ	0.93	1.07	0.76		0.92	
	PI3K inh.	0.67	1.04	0.64		0.69	
	[1]**TQ + PI3K inh.**	**0.59**	**0.53**	**0.47**		**0.62**	
[2]**Multiplication of TQxPI3K-Inh.**		**0.62**	**1.11**	**0.49**		**0.63**	
**Difference**[1]**-**[2]**at 4 h**		**−0.03**	**−0.59**	**−0.02**		**−0.01**	−0.16 (±0.28)**

RKO cells were treated with TQ, PI3K inhibitor or with a combination of both substances for 2 h or 4 h to test for syngergistic or additive effects on the dephosphorylation of GSK-3β. 5 different Western blots were performed and the relative ratios of p-GSK-3β and total GSK-3β protein levels were calculated using the program Image J. Mathematically, when effects are measured as proportions, the additive effect of two substances is the multiplication of the two values resulting from the single treatments. The mathematical product of both treatments is only minimally deviating from the actual values for the double treatment resulting from the Western blots. The null-hypothesis of no mean difference cannot be rejected at either time point (T-test, p-value *0.56 (2 h) and **0.34 (4 h)). While this result is in itself no proof of additivity, a closer inspection of the calculated differences shows that the assumption of additivity is indeed reasonable: In experiments 1, 3 and 5 the differences are very close to zero. Experiments 2 and 5 show stronger deviations, however in opposite directions and the resulting mean differences are close to zero for both time points.

We then investigated the RAS/RAF/MEK pathway, which also influences GSK-3β Ser9 phosphorylation. MEK1/2 inhibition with UO126 and concomitant incubation with TQ did not show additional reduction of GSK-3β phosphorylation (Figure 5E), assuming that both compounds are utilizing the same pathway. As stated above, pharmacological inhibition of MEK1/2 in RKO cells resulted in an abrogation of p-ERK1/2 phosphorylation that was not seen on treatment with TQ. Therefore, we assume that TQ carries out its function by inhibiting another kinase in this pathway.

## Discussion

**Figure 6:**
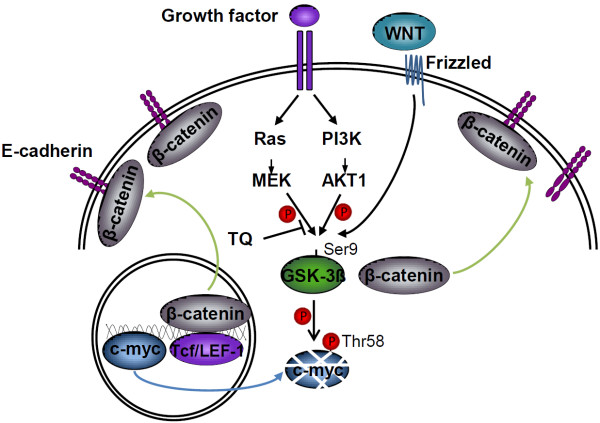
**A model of TQ’s effect on colon cancer cells.** In untreated colorectal cells GSK-3β is phosphorylated on Ser9 by various pathways (Ras-Raf-MEK, PI3K-AKT1, WNT), and thereby inactivated. This allows accumulation of β-catenin in the cytoplasm, its nuclear translocation and activation of Tcf/LEF-1. Thymoquinone (TQ) treatment reduces GSK-3β Ser9 phosphorylation (downstream of Ras, Raf, MEK) leading to a relocalization of β-catenin to the membrane (green arrows) and reduction of nuclear c-myc (likely via phosphorylation, ubiquitination and subsequent degradation; blue arrow; ref. 37).

In this study we demonstrate that TQ, the main active component in the essential oil of *Nigella sativa* seeds, reduces the number of large polyps in the small intestine of APC^Min^ mice. This effect was mainly due to induction of tumor cell-specific apoptosis. In addition, we found a shift of β-catenin to the membrane and a reduced nuclear c-myc expression in polyps. In RKO, TQ activates GSK-3β, which in turn might mediate phosphorylation and subsequent degradation of c-myc protein (model see Figure 6).

Black cumin seed oil is commonly used in traditional medicine and as a spice in countries with low prevalence for colorectal cancer [16]. It has been described to interfere with several tumor pathways. Gali-Muhtasib and others showed that TQ reduces the number of aberrant crypt foci and inhibits tumor growth in a 1,2-dimethylhydrazine-induced and a xenograft colon carcinogenesis mouse model, mainly by induction of apoptosis. Furthermore this group demonstrated that TQ decreases the invasive potential of a mouse colorectal cancer cell line [20]. Other publications showed that the antiproliferative and apoptotic effects of TQ are either mediated by p53-dependent or independent mechanisms. The former could be shown for colorectal cancer cells, the latter only for osteosarcoma cells [27] and a myeloblastic leukemia cell line [28]. p53-null HCT116 cells were less sensitive to TQ-induced growth arrest and apoptosis compared to p53-wt HCT116 cells [21]. The reduced apoptosis in p53^−/−^ HCT116 cells is due to an up-regulation of the DNA damage sensor CHK1, which is normally transcriptionally repressed by p53 [29]. This is in line with our observation that cells having functional p53, such as LoVo, HCEC-1CT, RKO and HCT116, are more sensitive to TQ induced cell death compared to cells with p53 mutations, as shown for DLD1 and HT29. Also, we could confirm in our model that one of the main chemopreventive pathways of TQ is the cancer cell-specific induction of apoptosis which is likely mediated by p53. TQ was shown to induce oxidative stress and subsequently H2AX phosphorylation in HCT116 cells, which was diminished in p53^−/−^ HCT116 [29]. The authors concluded that the changes in H2AX expression and the increased apoptosis correlated with enhanced mitochondrial ROS production. In addition to TQ’s tumor cell-specific induction of apoptosis we observed that TQ reverses the Ki-67 positivity found in the cells of the intermediate zone of APC^Min^ villi. This is indicative of lowered proliferation in the normal tissue. However, the number of Ki-67 was not altered in polyps. This result is in agreement with previous studies that assessed Ki-67 staining in HCT116 xenograft tumors [20].

*In vitro* analysis of TQ`s influence on the Ser9 phosphorylation status of GSK-3β, an important kinase for cell differentiation and apoptosis, showed reduced phosphorylation and activation of the protein. There is increasing evidence that c-myc Thr58 is phosphorylated by active GSK-3β, thereby regulating its stability by mediating Fbw7-driven turnover [26]. Enhanced GSK-3β activity by TQ might lead to phosphorylation and subsequent degradation of c-myc. The cause of reduced Ser9 phosphorylation in RKO cells is still elusive. Concomitant treatment with TQ and the PI3K inhibitor LY294002 leads to an additive reduction of p-GSK-3β, assuming that, in colon cells, TQ does not utilize the PI3K/AKT pathway, as shown for prostate cancer [18]. Instead, inhibition of MEK1/2 with UO126 reduced p-ERK1/2 and p-GSK-3β levels and concomitant treatment with TQ did not reveal an additive effect on GSK-3β phosphorylation, indicating that TQ mediates its effect over the RAS/RAF/MEK pathway rather than ERK1/2. As shown *in vivo* and *in vitro* TQ leads to membranous β-catenin translocation, which is probably also mediated by GSK-3β activation. This is in line with a study conducted by Li et. al. showing that active GSK-3β interacts with MUC1, thereby releasing MUC1 bound β-catenin, which then associates with membranous E-cadherin [30] (Figure 6).

One shortcoming of the study is the limited effect of TQ on tumor multiplicity. In this respect our results deviate from a chemically-induced colorectal cancer model [20]. TQ may have less activity in genetically-driven cancer models such as the APC^Min^ mouse than in chemically-induced models. Another contributing factor might be the administration route of TQ (as part of the chow versus intraperitoneal injections). Also differences in the mutational status of APC may partially explain this phenomenon. We have therefore used RKO cells (APC wt) for *in vitro* studies.

The TQ concentration in seeds is between 0.12 and 0.25% (w/v) [31,32]. In the essential oil, which is about 0.41 - 0.44% of the total seed, the TQ concentration is between 28 - 57% [31]. The human equivalent dose of 375 mg/kg TQ in the diet corresponds to 3.6 mg/kg body weight, resulting in a total amount of 219 mg for a body weight of 60 kg [33]. Studies using TQ in *H. pylori* infection [34] or diabetes [35] report an uptake of up to 3 g *Nigella sativa* seeds/day for one to three months, respectively. Both studies report amelioration of the disease but no severe side effects or adverse effects on renal or hepatic functions, respectively. Assuming a seeds content of 0.2% TQ, this results in an effective dose of 6 mg TQ per day. In mice, no adverse effects except for a decrease in glucose levels was observed with TQ concentrations up to 90 mg/kg body weight [36]. The corresponding concentration in our study was 45 mg/kg body weight for TQ-high. Thus the concentrations used are likely well tolerated without major toxicity. A dose dependent decrease of GSH levels in vital organs was found only with higher concentrations (2–3 g/kg, p.o.), most probably due to formation of TQ conjugates with GSH (glutathionyl-dihydrothymoquinone), which might affect bioavailability as well as antioxidant properties of TQ [36,37].

In summary, TQ interferes with polyp progression in APC^Min^ mice by inducing tumor cell-specific apoptosis and by modulating Wnt signaling through GSK-3β activation, β-catenin translocation and reduction of nuclear c-myc. TQ or *Nigella sativa* seed oil might be a useful nutritional supplement to complement current chemoprevention in FAP.

## Methods

### Mice, genotyping, treatment, and colonoscopy

Heterozygous male C57BL/6J-Apc^Min^/J (APC^Min^) mice (The Jackson Laboratory, Bar Harbor, ME) were bred with wild-type female C57BL/6J mice. Apc^Min^ mice harbor a nonsense mutation at nucleotide 2549 in the murine homolog of the human APC gene [38]. Genotyping was performed from mouse tail DNA using primer-introduced restriction analysis-polymerase chain reaction with APC primers (APC_for_: 5‘-TCT CGT TCT GAG AAA GAC AGA AGC T-3‘; APC_rev_: 5‘-TGA TAC TTC TTC CAA AGC TTT GGC TAT-3‘) and HindIII digestion according to Musteanu et.al [39]. 4–6 week old female and male APC^Min^ mice were housed at the Institute of Biomedical Research (Medical University Vienna, Vienna). Mice were kept under 12 hour light/dark cycles. Chow and water were available ad libitum. Animals were weighed weekly and the amount of food intake was documented. All animal experiments were performed in accordance with the Austrian and European law, defined by the Good Scientific Practice guidelines of the Medical University Vienna (animal ethics approval number: BMWF-66.009/0113-II/10b/2010).

The animals were randomly divided into 4 groups and treated over a period of 12 weeks. Chemopreventive substances were added to a commercial rodent diet (C1000, Altromin, Lage, Germany) as follows: 37.5 mg/kg chow of TQ (274666, Sigma Aldrich) referred to as TQ-low (n=13) and 375 mg/kg chow of TQ referred to as TQ-high (n=16). 200 mg/kg chow piroxicam (P5654, Sigma-Aldrich) was used as a positive control (n=15) [40,41]. Mice fed the diet alone served as a negative control (n=17). Mice gaining less than 1.5 g of weight were excluded for analysis, as the chemopreventive substance was taken up through the diet. Mice with breast cancer, which is associated with the APC^Min^ phenotype [42], were euthanized and excluded from analysis. At week 9 of treatment mice underwent colonoscopy. Briefly, mice were anesthetized with an i.p. injection of ketamine and xylazine, the colonoscope (Karl Storz, Tuttlingen, Germany) connected to an airpump (Eheim, Deizau, Germany) was inserted and the colon inflated [43]. During insertion and withdrawal of the colonoscope up to 3 cm the number of polyps was determined in real-time using a standard monitor and a video was recorded. Mice were euthanized, the intestine was dissected, flushed with PBS and 10% neutral buffered formalin, and coiled up to a Swiss roll [44]. Prior to paraffin embedding the intestine was fixed in neutral buffered formalin for 24 h.

### Histology, immunohistochemical analysis, and apoptosis

Serial tissue sections (4 μm) were H&E stained and analyzed by an expert pathologist (G.O.) who was blinded for the treatment group. Polyp number, localization (small intestine, colon), size (small < 0.3 mm; medium 0.3 – 1 mm; large polyp > 1 mm), and tumor grade (adenoma; adenocarcinoma – here defined by the penetration of the muscularis mucosae) were assessed [45]. Apoptosis was determined using the DeadEnd^TM^ Fluorometric TUNEL System (G3250, Promega, Mannheim, Germany). For visualization of nuclei and mounting Vectashield® Mounting Medium with DAPI (H-1200; Vector Laboratories, Burlingame, CA) was used and samples were analyzed by confocal microscopy (LSM 5 exciter; Zeiss Germany).

Immunohistochemical staining was performed on paraffin-embedded mouse intestine using antibodies against β-catenin, Ki-67, c-myc and isotype specific (anti-mouse/anti-rabbit) biotinylated secondary antibody and standard staining procedures (details for antibodies see Additional file 6: Table S1). Slides were dried, de-waxed in xylene and rehydrated using a decreasing alcohol series. After blocking of endogenous peroxidase with 15% H_2_0_2_ in methanol, antigen retrieval was performed in 10 mM citrate buffer, pH 6. Subsequently, slides were blocked in 2% horse serum, 3% BSA in TRIS buffer and endogenous IgG was blocked with Vector M.O.M. Blocking Reagent (MKB-2213, Vector Laboratories). Primary antibodies were incubated at 4°C overnight, followed by biotinylated secondary antibody and avidin-biotin-HRP complex (Vectastain ABC Kit, PK-6100; Vector Laboratories). Staining was visualized using 3,3′-diaminobenzidine (32750; Fluka) and nuclear counterstaining was performed using hematoxylin. Slides were dehydrated and embedded in Histofluid (6900002; Marienfeld, Lauda Koenigshofen, Germany). Images were recorded at 40× to 400× magnification using an Olympus BH-2 microscope and an Olympus E330 digital camera. Immunoreactivity was independently scored by two investigators. A standardized immunoreactivity scoring system was modified to evaluate both the intensity of immunohistochemical staining and the proportion of cells stained [46]. The staining intensity was classified into 0 (no staining), 1+ (weak), 2+ (moderate), 3+ (strong) and the percentage of positive cells was recorded (0-100%), resulting in a highest value of 300. Total counts were divided by 25 to reach a maximum immunoreactivity score (IRS) of 12, to be comparable to other publications using a scoring system reaching a maximum of 12 [46]. IRS was calculated for small polyps, large polyps and normal mucosa of 6 different fields of view (FoV). C-myc staining was evaluated by scoring the overall staining intensity per polyp (n=16) as follows: negative (0), weakly (1+) or moderately positive (2+).

### Cell culture and reagents

The human colon cancer cell lines (DLD1, HCT116, HT29, LoVo, and RKO), obtained from ATCC, were cultured in IMDM (Gibco/Invitrogen) supplemented with 10% FBS (Biochrom, Berlin, Germany) and Penicillin-Streptomycin solution (Gibco). The normal diploid human colon epithelial cell line HCEC (1CT) [47], a kind gift from Jerry W. Shay, were cultured as previously described [48]. Cells were incubated at 5% CO_2_, 37°C and a relative humidity of 95%. Cells were treated with 10–30 μM TQ (Sigma-Aldrich; 274666) and/or 30 μM PI3K inhibitor LY294002 (9901; NEB) or 30 μM MEK1/2 inhibitor UO126 (9903; NEB) for indicated times.

### Immunocytochemistry

Fluorescence immunocytochemistry was performed using antibodies against p-GSK-3β and GSK-3β. Cells were fixed, permeabilized, blocked and incubated with the primary antibody overnight at 4°C. For protein visualization secondary AlexaFluor 488 antibody was used (Additional file 6: Table S1). Nuclear counterstaining was performed using Vectashield mounting medium with DAPI. Images were scanned 400x magnification on a LSM 700 (Zeiss).

### Annexin V/propidium iodide staining

1.5×10^5^ RKO cells were seeded in a 6-well plate and grown for 24 h. Cells were treated with 0–120 μM TQ for 24 h or 5 μg/ml Actinomycin D (positive control) for 16 h and processed for Annexin V-FITC/propidium iodide (PI) staining according to the manufacturer’s protocol (eBioscience, BMS500FI). Annexin/PI positive cells were measured with a Cell Lab Quanta SC Flow Cytometer (Beckman Coulter) and analyzed with Quanta Analysis software.

### Cell viability assay

1.5×10^4^ cells/well were seeded in a 96-well microtiter plate and grown for 24 h. After a 24 h-treatment with 0–500 μM TQ, cells were incubated for 3 h with 0.5 mg/mL MTT (Sigma, M5655). The cells were subjected to a 1:1 ethanol/DMSO treatment to dissolve formazan crystals. The intensity of the solubilized crystals was measured colorimetrically at 570 nm (Anthos 2010). Each measurement was performed in biological quadruplicates.

### Western blotting

Cells were rinsed with PBS, lysed in ice-cold RIPA buffer, and centrifuged. The protein concentration was determined via Bradford assay and equal protein amounts (25 μg) were boiled in SDS gel sample buffer. Proteins were separated by SDS–PAGE and immunoblotted onto a PVDF membrane. Nuclear and cytoplasmic separation was carried out as published elsewhere [49]. Membranous and cytoplasmic separation was performed as described by Howard et al. [50]. Primary antibodies were used as follows: β-catenin, p-GSK-3β (Ser9), GSK-3β, c-myc, p-ERK1/2 (Thr202/Tyr204), ERK1/2, p-Akt (Ser473), Akt, α-tubulin, Na-K-ATPase, and fibrillarin (Additional file 6: Table S1). Bands were visualized with anti-rabbit or anti-mouse IRDye coupled antibodies and scanned on Odyssey imager (LI-COR). Densitometry was done with ImageJ 1.45 (http://rsb.info.nih.gov/ij/).

### Quantitative real time-PCR

Total RNA was isolated with TRIZOL reagent (Biorad) and was reverse transcribed to cDNA using the Thermoscript RT-PCR System (11146–024; Invitrogen) according to the manufacturer’s protocols. Quantitative real time-PCR was carried out in duplicates using Fast SYBR Green Master Mix (AB; 4385612) and c-myc primers (Additional file 6: Table S2). Data were normalized to two endogenous controls GAPDH and β-actin (QIAGEN). Relative expression levels of the transcripts were calculated using the comparative CT method [51].

### Statistics

Statistical analysis was performed using SPSS software version 17.0. Polyp number, size, and the number of apoptotic cells (TUNEL assay) were analyzed using univariate analysis of variance (ANOVA). Results were corrected by Dunnett (2-sided). Paired T-test was used to analyze the number of apoptotic cells in the normal mucosa compared to neoplastic tissue within one group. Immunohistochemistry data for β-catenin, Ki-67 and c-myc staining were analyzed using ANOVA. Results were corrected by Dunnett (2-sided). To investigate the additive or synergistic nature of the effects of TQ and PI3K-inhibitor on GSK-3β dephosphorylation the expected effects under the assumption of additivity were compared to the actual effects measured in the double treatment experiments. Since the effects are measured as proportions, an additive nature of effects means that the expected double effect is the product of the single effects. This product was calculated for each experiment and point in time and compared to the measured effect of the double treatment by calculating the difference. The resulting differences are reported for all experiments. The mean and the standard deviation of the differences were calculated for 2 h and 4 h points in time. Paired T-tests were performed to test the null-hypotheses of the mean differences being zero. p-values were considered as statistical significant if less than 0.05 (*p<0.05; **p<0.01; ***p<0.001). Data are expressed by mean and the 95% confidence interval for the mean (95% CI) or mean and standard deviation.

## Abbreviations

APC: Adenomatous polyposis coli gene; FAP: Familial adenomatous polyposis; FoV: Field of view; IRS: Immunoreactivity score; TQ: Thymoquinone.

## Competing interest

The authors disclose no conflicts.

## Authors’ contributions

ML: Conception and design, acquisition of data, analysis and interpretation of data, writing of the manuscript, MB: Acquisition of data, analysis and interpretation of data. GO: Analysis and interpretation of data. RE: Statistical analysis. KJ: Acquisition of data, analysis and interpretation of data, proof-reading of the manuscript. KWD: Development of methodology, revision of the manuscript. MJ: Technical support, analysis and interpretation of data. VK: Conception and design, interpretation of data, revision of the manuscript. CC: Conception and design, revision of the manuscript. RR: Statistical analysis. CG: Obtained funding, conception and design, study supervision, revision of the manuscript. All authors read and approved the final manuscript.

## Supplementary Material

Additional file 1: Figure S1 Average weight curves [g] of female (**A**) and male (**B**) APC^Min^ mice treated with TQ-low (n_female_=8, n_male_=5 dashed line), TQ-high (n_female_=10 n_male_=6 , dots), piroxicam (n_female_=11, n_male_=4 dot-dashed line) or left untreated (n_female_=12, n_male_=5 , line). Representative H&E images of tumors with different size: (I) <0.3 mm/small, (II) 0.3-1 mm/medium, (III) >1 mm/large; 40x (**C**). Average food intake in grams per mouse a day for each treatment group (**D**). H&E images of the two adenocarcinomas, defined by penetration of the muscularis mucosae, (arrows) found in the small intestine in the TQ-low (I) and TQ-high (II) treated group; 100× (**E**). Click here for file

Additional file 2: Figure S2 Colonoscopy and small intestinal tumor number. Number of polyps/mouse detected during colonoscopy after 9 weeks of treatment, reaching 2–3 cm into the colon (**A**). Representative images of normal mucosa (I) and polyps of different size (II-IV) are shown (**B**). For TQ-low (n=14) and piroxicam (n=14) a significantly reduced number of polyps compared to untreated (n=17) mice was found via colonoscopy. For TQ-high (n=17) a trend for reduction of polyps was seen. Every dot represents the number of polyps of a single mouse. *p<0.05; ANOVA, Dunnett 2-sided. Total number of polyps in the SI of APC^Min^ mice (**C**). Bars show mean number (± SD) of SI polyps/mouse. Piroxicam decreased the total number of SI polyps. For TQ-high there was a trend for reduction of total SI polyps. ***p<0.001; ANOVA, Dunnett 2-sided. Click here for file

Additional file 3: Figure S3 Proliferation. Ki-67 IRS (**A**) calculation for villi, crypts and polyps (n=8 each, 4 mice) of untreated (**B**), piroxicam (**C**), TQ-low (**D**) and TQ-high (**E**) treated APC^Min^ mice. Bar graphs show mean Ki-67 IRSs (± SD). High dose TQ lowered the amount of Ki-67 positive cells in the villi compared to control samples, for low dose TQ there was a trend to that effect (**A**). Representative images of Ki-67 staining in the small intestine (**B**-**E**); upper left panel: normal mucosa; upper right panel: polyp; lower panel: magnification of a single villus. Panel F shows the negative control sample where the primary antibody was omitted (**F**). *p<0.05; ANOVA, Dunnett, 2-sided was used to compare the different treatment groups to the control group. Magnification: 100× (top panel), 400× (bottom panel). Click here for file

Additional file 4: Figure S4 MTT assay. TQ exerts different effects on cell viability in cancer cell lines having various mutational backgrounds. Colon cancer cells and 1CT normal diploid human colon epithelial cells were incubated with TQ at different concentrations in quadruplicates for 24 h. The absorbance values (relative OD), expressed as means, compared to untreated cells were measured by MTT assay and IC50 concentrations were calculated (**A**). TQ does not exert its effect on the p-ERK1/2 and p-AKT1 (Ser473) pathway, but inhibition of MEK1/2 with the compound UO126 at 30 μM abrogated p-ERK1/2 phosphorylation (Thr202/Tyr204) between 30’ and 12 h and reduced to a much lower extent p-AKT (Ser473), shown for whole cell lysates of RKO cells (**B** and **D**). Colon cancer cell lines RKO and HT29 were treated with 30 μM TQ for indicated times or left untreated (control). C-myc transcript levels stay constant upon TQ treatment. Relative mRNA expression levels of c-myc were calculated with GAPDH and β-actin as endogenous controls using qRT-PCR and the ΔΔct method. Error bars represent standard deviations of technical duplicates (**C**). Click here for file

Additional file 5: Figure S5 Annexin V/propidium iodide staining. RKO cells were treated with increasing concentrations of TQ for 24 h (**A**-**E**), with 5 μg/ml actinomycin D (positive control) for 16 h (**F**) or with the DMSO (solvent for TQ) for 24 h (**G**). The number of viable (lower left quadrant), early apoptotic (lower right quadrant), apoptotic (upper right quadrant) and dead cells (upper left quadrant) was measured on a Cell Lab Quanta SC Flow Cytometer and calculated with the Quanta Analysis software (**H**). With increasing TQ concentrations the number of apoptotic and dead cells is enriched. Click here for file

Additional file 6: Table S1 Antibody list; *Immunohistochemistry; **Immunocytochemistry. **Table S2.** qRT-PCR primer list. Click here for file

## References

[B1] Ferlay J Shin HR Bray F Forman D Mathers C Parkin DM Estimates of worldwide burden of cancer in 2008: GLOBOCAN 2008 Int J Cancer 2010 127 2893 2917 10.1002/ijc.25516 21351269

[B2] Boyle P Levin B Boyle P, Levin B 5.7 Colorectal Cancer World Cancer Report 2008 2008 1 Lyon: International Agency for Research on Cancer (IARC) 374 379

[B3] Nishisho I Nakamura Y Miyoshi Y Miki Y Ando H Horii A Koyama K Utsunomiya J Baba S Hedge P Mutations of chromosome 5q21 genes in FAP and colorectal cancer patients Science 1991 253 665 669 10.1126/science.1651563 1651563

[B4] Groden J Thliveris A Samowitz W Carlson M Gelbert L Albertsen H Joslyn G Stevens J Spirio L Robertson M Identification and characterization of the familial adenomatous polyposis coli gene Cell 1991 66 589 600 10.1016/0092-8674(81)90021-0 1651174

[B5] Burn J Bishop DT Chapman PD Elliott F Bertario L Dunlop MG Eccles D Ellis A Evans DG Fodde R A randomized placebo-controlled prevention trial of aspirin and/or resistant starch in young people with familial adenomatous polyposis Cancer Prev Res (Phila) 2011 4 655 665 10.1158/1940-6207.CAPR-11-0106 21543343 PMC3092423

[B6] Cruz-Correa M Hylind LM Romans KE Booker SV Giardiello FM Long-term treatment with sulindac in familial adenomatous polyposis: a prospective cohort study Gastroenterology 2002 122 641 645 10.1053/gast.2002.31890 11874996

[B7] Giardiello FM Yang VW Hylind LM Krush AJ Petersen GM Trimbath JD Piantadosi S Garrett E Geiman DE Hubbard W Primary chemoprevention of familial adenomatous polyposis with sulindac N Engl J Med 2002 346 1054 1059 10.1056/NEJMoa012015 11932472 PMC2225537

[B8] Steinbach G Lynch PM Phillips RK Wallace MH Hawk E Gordon GB Wakabayashi N Saunders B Shen Y Fujimura T The effect of celecoxib, a cyclooxygenase-2 inhibitor, in familial adenomatous polyposis N Engl J Med 2000 342 1946 1952 10.1056/NEJM200006293422603 10874062

[B9] Phillips RK Wallace MH Lynch PM Hawk E Gordon GB Saunders BP Wakabayashi N Shen Y Zimmerman S Godio L A randomised, double blind, placebo controlled study of celecoxib, a selective cyclooxygenase 2 inhibitor, on duodenal polyposis in familial adenomatous polyposis Gut 2002 50 857 860 10.1136/gut.50.6.857 12010890 PMC1773237

[B10] Arber N Spicak J Racz I Zavoral M Breazna A Gerletti P Lechuga MJ Collins N Rosenstein RB Eagle CJ Five-year analysis of the prevention of colorectal sporadic adenomatous polyps trial Am J Gastroenterol 2011 106 1135 1146 10.1038/ajg.2011.116 21503000

[B11] Solomon SD McMurray JJ Pfeffer MA Wittes J Fowler R Finn P Anderson WF Zauber A Hawk E Bertagnolli M Cardiovascular risk associated with celecoxib in a clinical trial for colorectal adenoma prevention N Engl J Med 2005 352 1071 1080 10.1056/NEJMoa050405 15713944

[B12] Zell JA Pelot D Chen WP McLaren CE Gerner EW Meyskens FL Risk of cardiovascular events in a randomized placebo-controlled, double-blind trial of difluoromethylornithine plus sulindac for the prevention of sporadic colorectal adenomas Cancer Prev Res (Phila) 2009 2 209 212 10.1158/1940-6207.CAPR-08-0203 19258540 PMC2739681

[B13] Kim B Giardiello FM Chemoprevention in familial adenomatous polyposis Best Pract Res Clin Gastroenterol 2011 25 607 622 10.1016/j.bpg.2011.08.002 22122775 PMC3569729

[B14] Fini L Piazzi G Daoud Y Selgrad M Maegawa S Garcia M Fogliano V Romano M Graziani G Vitaglione P Chemoprevention of intestinal polyps in ApcMin/+ mice fed with western or balanced diets by drinking annurca apple polyphenol extract Cancer Prev Res (Phila) 2011 4 907 915 10.1158/1940-6207.CAPR-10-0359 21383028 PMC3793841

[B15] Rajput S Mandal M Antitumor promoting potential of selected phytochemicals derived from spices: a review Eur J Cancer Prev 2012 21 205 215 10.1097/CEJ.0b013e32834a7f0c 21876437

[B16] Woo CC Kumar AP Sethi G Tan KH Thymoquinone: potential cure for inflammatory disorders and cancer Biochem Pharmacol 2012 83 443 451 10.1016/j.bcp.2011.09.029 22005518

[B17] Li F Rajendran P Sethi G Thymoquinone inhibits proliferation, induces apoptosis and chemosensitizes human multiple myeloma cells through suppression of signal transducer and activator of transcription 3 activation pathway Br J Pharmacol 2010 161 541 554 10.1111/j.1476-5381.2010.00874.x 20880395 PMC2990154

[B18] Yi T Cho SG Yi Z Pang X Rodriguez M Wang Y Sethi G Aggarwal BB Liu M Thymoquinone inhibits tumor angiogenesis and tumor growth through suppressing AKT and extracellular signal-regulated kinase signaling pathways Mol Cancer Ther 2008 7 1789 1796 10.1158/1535-7163.MCT-08-0124 18644991 PMC2587125

[B19] Das S Dey KK Dey G Pal I Majumder A MaitiChoudhury S Kundu SC Mandal M Antineoplastic and apoptotic potential of traditional medicines thymoquinone and diosgenin in squamous cell carcinoma PLoS One 2012 7 e46641 10.1371/journal.pone.0046641 23077516 PMC3471895

[B20] Gali-Muhtasib H Ocker M Kuester D Krueger S El-Hajj Z Diestel A Evert M El-Najjar N Peters B Jurjus A Thymoquinone reduces mouse colon tumor cell invasion and inhibits tumor growth in murine colon cancer models J Cell Mol Med 2008 12 330 342 18366456 10.1111/j.1582-4934.2007.00095.x PMC3823493

[B21] Gali-Muhtasib H Diab-Assaf M Boltze C Al-Hmaira J Hartig R Roessner A Schneider-Stock R Thymoquinone extracted from black seed triggers apoptotic cell death in human colorectal cancer cells via a p53-dependent mechanism Int J Oncol 2004 25 857 866 15375533

[B22] Dang CV MYC on the path to cancer Cell 2012 149 22 35 10.1016/j.cell.2012.03.003 22464321 PMC3345192

[B23] Klaus A Birchmeier W Wnt signalling and its impact on development and cancer Nat Rev Cancer 2008 8 387 398 10.1038/nrc2389 18432252

[B24] da Costa LT He TC Yu J Sparks AB Morin PJ Polyak K Laken S Vogelstein B Kinzler KW CDX2 is mutated in a colorectal cancer with normal APC/beta-catenin signaling Oncogene 1999 18 5010 5014 10.1038/sj.onc.1202872 10490837

[B25] Baker SJ Markowitz S Fearon ER Willson JK Vogelstein B Suppression of human colorectal carcinoma cell growth by wild-type p53 Science 1990 249 912 915 10.1126/science.2144057 2144057

[B26] Welcker M Orian A Jin J Grim JE Harper JW Eisenman RN Clurman BE The Fbw7 tumor suppressor regulates glycogen synthase kinase 3 phosphorylation-dependent c-Myc protein degradation Proc Natl Acad Sci USA 2004 101 9085 9090 10.1073/pnas.0402770101 15150404 PMC428477

[B27] Roepke M Diestel A Bajbouj K Walluscheck D Schonfeld P Roessner A Schneider-Stock R Gali-Muhtasib H Lack of p53 augments thymoquinone-induced apoptosis and caspase activation in human osteosarcoma cells Cancer Biol Ther 2007 6 160 169 10.4161/cbt.6.2.3575 17218778

[B28] El-Mahdy MA Zhu Q Wang QE Wani G Wani AA Thymoquinone induces apoptosis through activation of caspase-8 and mitochondrial events in p53-null myeloblastic leukemia HL-60 cells Int J Cancer 2005 117 409 417 10.1002/ijc.21205 15906362

[B29] Gali-Muhtasib H Kuester D Mawrin C Bajbouj K Diestel A Ocker M Habold C Foltzer-Jourdainne C Schoenfeld P Peters B Thymoquinone triggers inactivation of the stress response pathway sensor CHEK1 and contributes to apoptosis in colorectal cancer cells Cancer Res 2008 68 5609 5618 10.1158/0008-5472.CAN-08-0884 18632613

[B30] Li Y Bharti A Chen D Gong J Kufe D Interaction of glycogen synthase kinase 3beta with the DF3/MUC1 carcinoma-associated antigen and beta-catenin Mol Cell Biol 1998 18 7216 7224 9819408 10.1128/mcb.18.12.7216 PMC109303

[B31] Houghton PJ Zarka R HB Dl Hoult JR Fixed oil of Nigella sativa and derived thymoquinone inhibit eicosanoid generation in leukocytes and membrane lipid peroxidation Planta Med 1995 61 33 36 10.1055/s-2006-957994 7700988

[B32] Burits M Bucar F Antioxidant activity of Nigella sativa essential oil Phytother Res 2000 14 323 328 10.1002/1099-1573(200008)14:5<323::AID-PTR621>3.0.CO;2-Q 10925395

[B33] Reagan-Shaw S Nihal M Ahmad N Dose translation from animal to human studies revisited FASEB J 2008 22 659 661 17942826 10.1096/fj.07-9574LSF

[B34] Salem EM Yar T Bamosa AO Al-Quorain A Yasawy MI Alsulaiman RM Randhawa MA Comparative study of Nigella Sativa and triple therapy in eradication of Helicobacter Pylori in patients with non-ulcer dyspepsia Saudi J Gastroenterol 2010 16 207 214 10.4103/1319-3767.65201 20616418 PMC3003218

[B35] Bamosa AO Kaatabi H Lebdaa FM Elq AM Al-Sultanb A Effect of Nigella sativa seeds on the glycemic control of patients with type 2 diabetes mellitus Indian J Physiol Pharmacol 2010 54 344 354 21675032

[B36] Badary OA Al-Shabanah OA Nagi MN Al-Bekairi AM Elmazar MMA Acute and subchronic toxicity of thymoquinone in mice Drug Dev Res 1998 44 56 61 10.1002/(SICI)1098-2299(199806/07)44:2/3<56::AID-DDR2>3.0.CO;2-9

[B37] Khalife KH Lupidi G Reduction of hypervalent states of myoglobin and hemoglobin to their ferrous forms by thymoquinone: the role of GSH, NADH and NADPH Biochim Biophys Acta 2008 1780 627 637 10.1016/j.bbagen.2007.12.006 18206117

[B38] Su LK Kinzler KW Vogelstein B Preisinger AC Moser AR Luongo C Gould KA Dove WF Multiple intestinal neoplasia caused by a mutation in the murine homolog of the APC gene Science 1992 256 668 670 10.1126/science.1350108 1350108

[B39] Musteanu M Blaas L Mair M Schlederer M Bilban M Tauber S Esterbauer H Mueller M Casanova E Kenner L Stat3 is a negative regulator of intestinal tumor progression in Apc(Min) mice Gastroenterology 2010 138 1003 1011 10.1053/j.gastro.2009.11.049 19962983

[B40] Ritland SR Gendler SJ Chemoprevention of intestinal adenomas in the ApcMin mouse by piroxicam: kinetics, strain effects and resistance to chemosuppression Carcinogenesis 1999 20 51 58 10.1093/carcin/20.1.51 9934849

[B41] Jacoby RF Marshall DJ Newton MA Novakovic K Tutsch K Cole CE Lubet RA Kelloff GJ Verma A Moser AR Chemoprevention of spontaneous intestinal adenomas in the Apc Min mouse model by the nonsteroidal anti-inflammatory drug piroxicam Cancer Res 1996 56 710 714 8631000

[B42] Moser AR Mattes EM Dove WF Lindstrom MJ Haag JD Gould MN ApcMin, a mutation in the murine Apc gene, predisposes to mammary carcinomas and focal alveolar hyperplasias Proc Natl Acad Sci USA 1993 90 8977 8981 10.1073/pnas.90.19.8977 8415640 PMC47484

[B43] Becker C Fantini MC Wirtz S Nikolaev A Kiesslich R Lehr HA Galle PR Neurath MF In vivo imaging of colitis and colon cancer development in mice using high resolution chromoendoscopy Gut 2005 54 950 954 10.1136/gut.2004.061283 15951540 PMC1774595

[B44] Moolenbeek C Ruitenberg EJ The "Swiss roll": a simple technique for histological studies of the rodent intestine Lab Anim 1981 15 57 59 10.1258/002367781780958577 7022018

[B45] Boivin GP Washington K Yang K Ward JM Pretlow TP Russell R Besselsen DG Godfrey VL Doetschman T Dove WF Pathology of mouse models of intestinal cancer: consensus report and recommendations Gastroenterology 2003 124 762 777 10.1053/gast.2003.50094 12612914

[B46] Lopez DS I Fan J Yang X Zonderman AB Potapova O Pizer ES Gorospe M Role of the RNA-binding protein HuR in colon carcinogenesis Oncogene 2003 22 7146 7154 10.1038/sj.onc.1206862 14562043

[B47] Roig AI Eskiocak U Hight SK Kim SB Delgado O Souza RF Spechler SJ Wright WE Shay JW Immortalized epithelial cells derived from human colon biopsies express stem cell markers and differentiate in vitro Gastroenterology 2010 138 1012 1021 10.1053/j.gastro.2009.11.052 19962984

[B48] Campregher C Schmid G Ferk F Knasmuller S Khare V Kortum B Dammann K Lang M Scharl T Spittler A MSH3-Deficiency Initiates EMAST without Oncogenic Transformation of Human Colon Epithelial Cells PLoS One 2012 7 e50541 10.1371/journal.pone.0050541 23209772 PMC3507781

[B49] Lee JH Kang Y Khare V Jin ZY Kang MY Yoon Y Hyun JW Chung MH Cho SI Jun JY The p53-inducible gene 3 (PIG3) contributes to early cellular response to DNA damage Oncogene 2010 29 1431 1450 10.1038/onc.2009.438 20023697

[B50] Howard S Deroo T Fujita Y Itasaki N A positive role of cadherin in Wnt/beta-catenin signalling during epithelial-mesenchymal transition PLoS One 2011 6 e23899 10.1371/journal.pone.0023899 21909376 PMC3166074

[B51] Pfaffl MW A new mathematical model for relative quantification in real-time RT-PCR Nucleic Acids Res 2001 29 e45 10.1093/nar/29.9.e45 11328886 PMC55695

