# Supplementary figures and images for: Thymoquinone attenuates tumor growth in ApcMin mice by interference with Wnt-signaling
Source: Mol Cancer. 2013 May 13;12:41. doi: 10.1186/1476-4598-12-41 (PMC3663767; doi:10.1186/1476-4598-12-41)

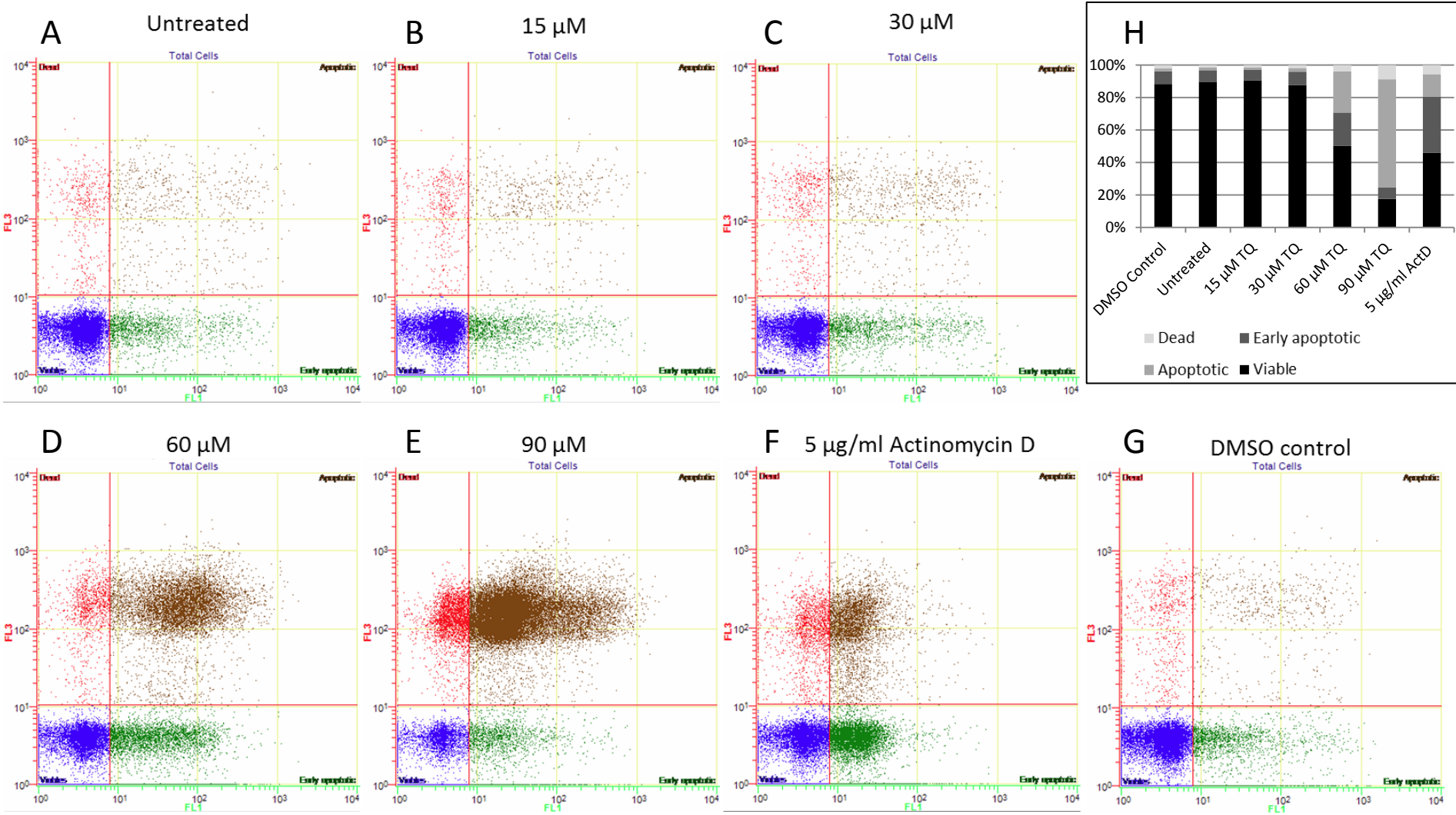

Supplement: Additional file 1: Figure S1 — Average weight curves [g] of female (A) and male (B) APCMin mice treated with TQ-low (nfemale=8, nmale=5 dashed line), TQ-high (nfemale=10 nmale=6 , dots), piroxicam (nfemale=11, nmale=4 dot-dashed line) or left untreated (nfemale=12, nmale=5 , line). Representative H&E images of tumors with different size: (I) <0.3 mm/small, (II) 0.3-1 mm/medium, (III) >1 mm/large; 40x (C). Average food intake in grams per mouse a day for each treatment group (D). H&E images of the two adenocarcinomas, defined by penetration of the muscularis mucosae, (arrows) found in the small intestine in the TQ-low (I) and TQ-high (II) treated group; 100× (E). [file 1476-4598-12-41-S1.pdf]

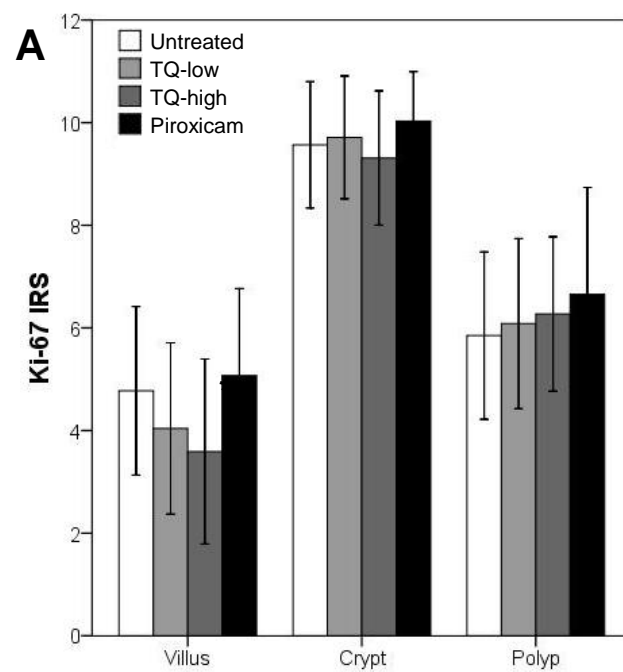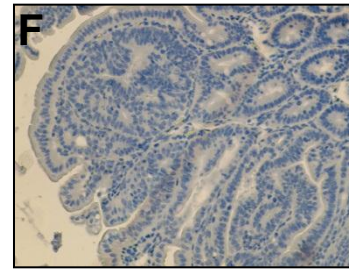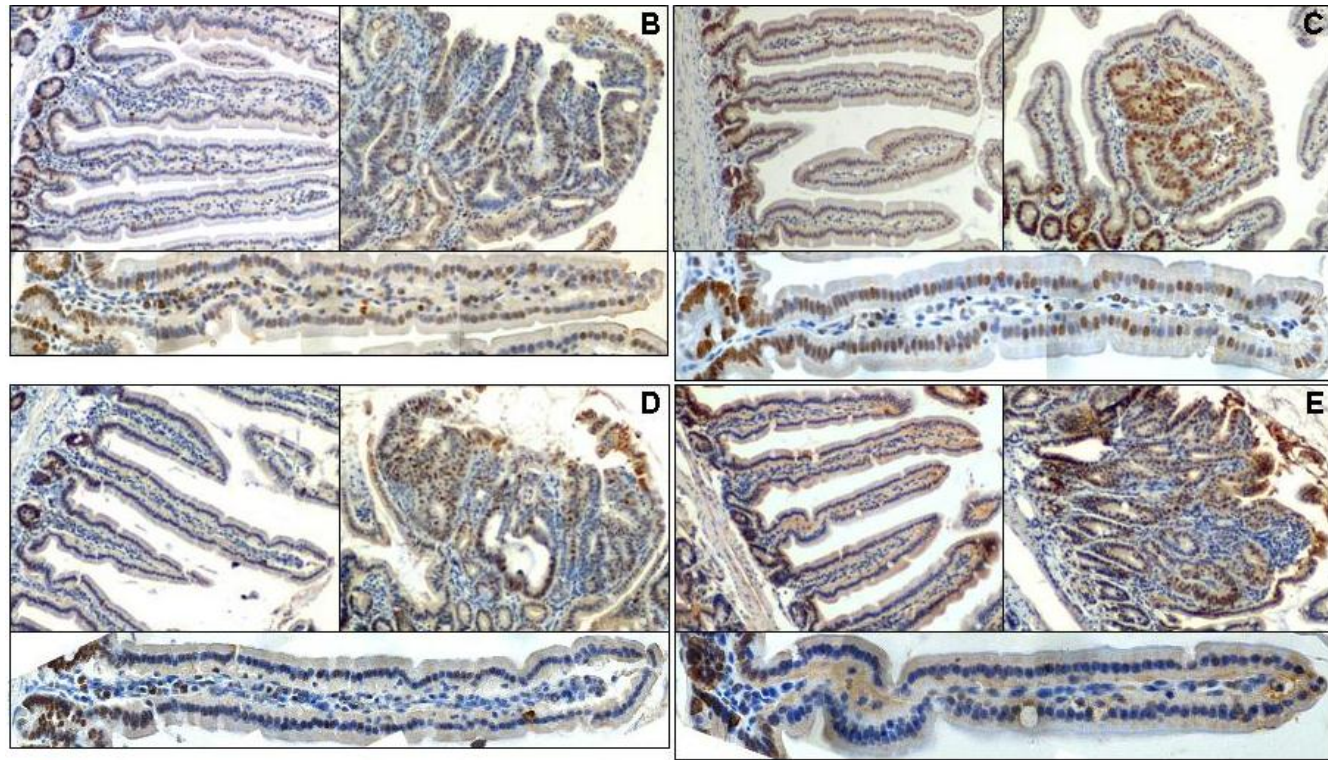

Supplement: Additional file 3: Figure S3 — Proliferation. Ki-67 IRS (A) calculation for villi, crypts and polyps (n=8 each, 4 mice) of untreated (B), piroxicam (C), TQ-low (D) and TQ-high (E) treated APCMin mice. Bar graphs show mean Ki-67 IRSs (± SD). High dose TQ lowered the amount of Ki-67 positive cells in the villi compared to control samples, for low dose TQ there was a trend to that effect (A). Representative images of Ki-67 staining in the small intestine (B-E); upper left panel: normal mucosa; upper right panel: polyp; lower panel: magnification of a single villus. Panel F shows the negative control sample where the primary antibody was omitted (F). *p<0.05; ANOVA, Dunnett, 2-sided was used to compare the different treatment groups to the control group. Magnification: 100× (top panel), 400× (bottom panel). [file 1476-4598-12-41-S3.pdf]

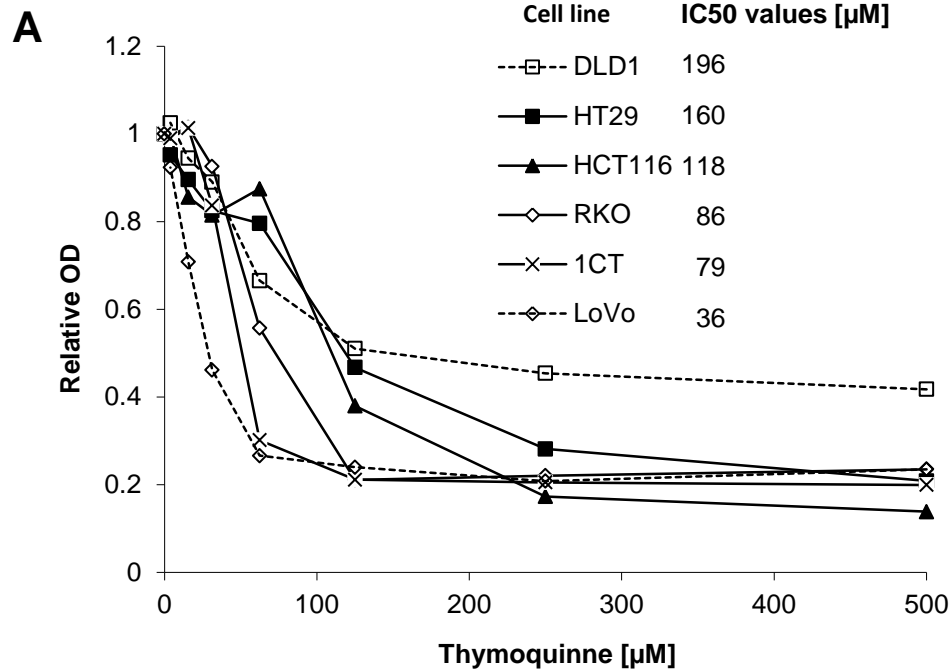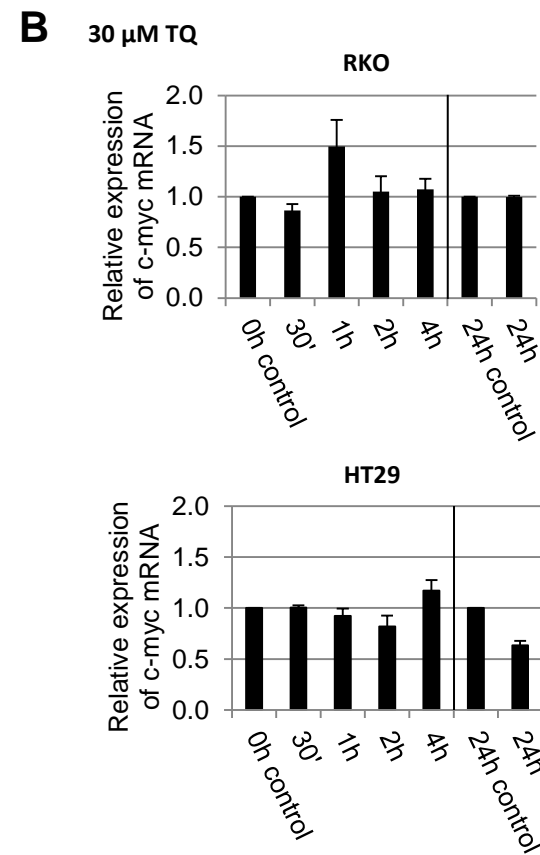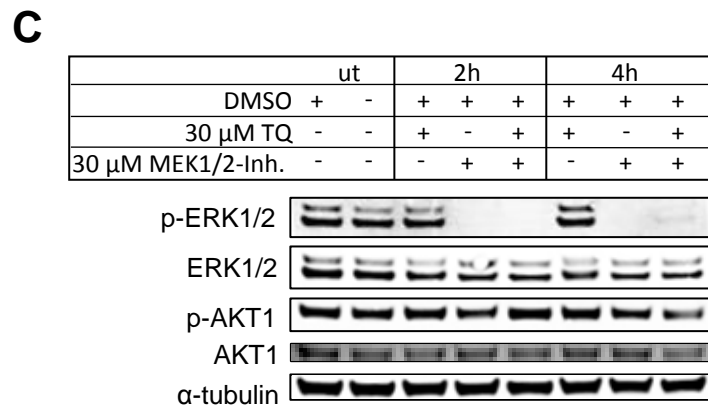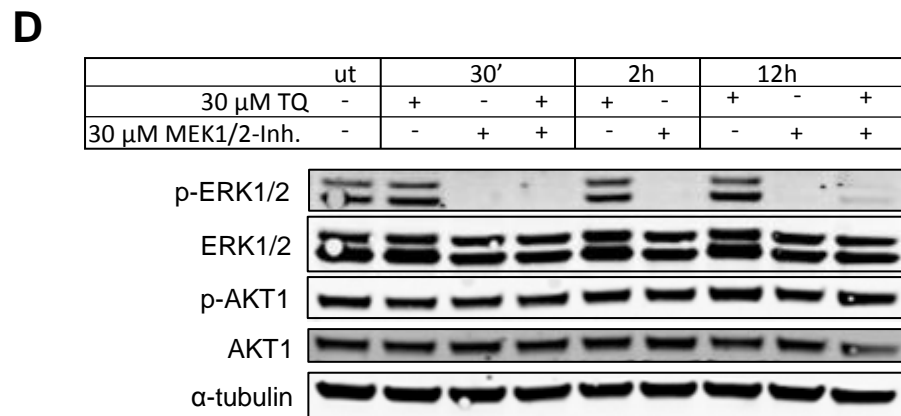

Supplement: Additional file 4: Figure S4 — MTT assay. TQ exerts different effects on cell viability in cancer cell lines having various mutational backgrounds. Colon cancer cells and 1CT normal diploid human colon epithelial cells were incubated with TQ at different concentrations in quadruplicates for 24 h. The absorbance values (relative OD), expressed as means, compared to untreated cells were measured by MTT assay and IC50 concentrations were calculated (A). TQ does not exert its effect on the p-ERK1/2 and p-AKT1 (Ser473) pathway, but inhibition of MEK1/2 with the compound UO126 at 30 μM abrogated p-ERK1/2 phosphorylation (Thr202/Tyr204) between 30’ and 12 h and reduced to a much lower extent p-AKT (Ser473), shown for whole cell lysates of RKO cells (B and D). Colon cancer cell lines RKO and HT29 were treated with 30 μM TQ for indicated times or left untreated (control). C-myc transcript levels stay constant upon TQ treatment. Relative mRNA expression levels of c-myc were calculated with GAPDH and β-actin as endogenous controls using qRT-PCR and the ΔΔct method. Error bars represent standard deviations of technical duplicates (C). [file 1476-4598-12-41-S4.pdf]
