# Supplementary material for: Thymoquinone attenuates tumor growth in ApcMin mice by interference with Wnt-signaling
Source: Mol Cancer. 2013 May 13;12:41. doi: 10.1186/1476-4598-12-41 (PMC3663767; doi:10.1186/1476-4598-12-41)
Supplement: Additional file 2: Figure S2 — Colonoscopy and small intestinal tumor number. Number of polyps/mouse detected during colonoscopy after 9 weeks of treatment, reaching 2–3 cm into the colon (A). Representative images of normal mucosa (I) and polyps of different size (II-IV) are shown (B). For TQ-low (n=14) and piroxicam (n=14) a significantly reduced number of polyps compared to untreated (n=17) mice was found via colonoscopy. For TQ-high (n=17) a trend for reduction of polyps was seen. Every dot represents the number of polyps of a single mouse. *p<0.05; ANOVA, Dunnett 2-sided. Total number of polyps in the SI of APCMin mice (C). Bars show mean number (± SD) of SI polyps/mouse. Piroxicam decreased the total number of SI polyps. For TQ-high there was a trend for reduction of total SI polyps. ***p<0.001; ANOVA, Dunnett 2-sided. [file 1476-4598-12-41-S2.pdf]

Suppl. Table 1.

Antibody	Dilution	Application	Company	Catalog number
β-catenin	1:500	IHC*	BD Transduction Laboratories	610153
Ki-67	1:500	IHC	Abcam	ab15580
c-myc	1:1500	IHC	Abcam	ab39688
Biotinylated horse anti-mouse IgG antibody	1:100	IHC (2ndary)	Vector Laboratories	BA-2000
Biotinylated goat anti-rabbit IgG antibody	1:100	IHC (2ndary)	Vector Laboratories	BA-1000
β-catenin	1:2000	Western blot	BD Transduction Laboratories	610153
p-GSK-3β (serine 9)	1:1000	Western blot	Cell Signaling	9336
GSK3-3β	1:5000	Western blot	Abcam	ab32391
c-myc	1:1000	Western blot	Cell Signaling	5605
p-ERK1/2 (Thr202/Tyr204)	1:2000	Western blot	Cell Signaling	9106
ERK1/2	1:1000	Western blot	Cell Signaling	4695
p-AKT1 (Ser 473)	1:2000	Western blot	Cell Signaling	4060
AKT1 (pan, 40D4)	1:2000	Western blot	Cell Signaling	2920
α-tubulin	1:10000	Western blot	Abcam	ab7291
Na-K-ATPase	1:1000	Western blot	Cell Signaling	3010
fibrillarin	1:1000	Western blot	Abcam	ab5821
IRDye® 680 donkey anti-mouse IgG (H+L)	1:30000	Western blot (2ndary)	LI-COR	926-32222
IRDye® 800CW goat anti-rabbit IgG (H+L)	1:30000	Western blot (2ndary)	LI-COR	926-32211
p-GSK-3β (serine 9)	1:250	ICC**	Cell Signaling	9336
GSK3-3β	1:250	ICC	Abcam	ab32391
Alexa Fluor® 488 goat anti-rabbit IgG (H+L)	1:500	ICC (2ndary)	Invitrogen	A-11008

Suppl. Table 2.

Primer	Sequence
c-myc$_{for}$	5'-GCT GCT TAG ACG CTG GAT TT-3'
c-myc$_{rev}$	5'-CAC CGA GTC GTA GTC GAG GT-3'
β-actin	Hs_ACTB_2_SG (QT01680476) - Qiagen
GAPDH	Hs_GAPDH_2_SG (QT01192646) - Qiagen
